# Atrial-ventricular differences in rabbit cardiac voltage-gated Na^+^ currents: Basis for atrial-selective block by ranolazine

**DOI:** 10.1016/j.hrthm.2017.06.012

**Published:** 2017-11

**Authors:** Rachel E. Caves, Hongwei Cheng, Stéphanie C. Choisy, Hanne C. Gadeberg, Simon M. Bryant, Jules C. Hancox, Andrew F. James

**Affiliations:** School of Physiology, Pharmacology & Neuroscience, University of Bristol, Bristol, United Kingdom

**Keywords:** Antiarrhythmic drug, Atrial myocytes, Cardiac regional heterogeneity, Na^+^ channel blocker, Ventricular myocytes

## Abstract

**Background:**

Class 1 antiarrhythmic drugs are highly effective in restoring and maintaining sinus rhythm in atrial fibrillation patients but carry a risk of ventricular tachyarrhythmia. The antianginal agent ranolazine is a prototypic atrial-selective voltage-gated Na^+^ channel blocker but the mechanisms underlying its atrial-selective action remain unclear.

**Objective:**

The present study examined the mechanisms underlying the atrial-selective action of ranolazine.

**Methods:**

Whole-cell voltage-gated Na^+^ currents (*I*_Na_) were recorded at room temperature (∼22°C) from rabbit isolated left atrial and right ventricular myocytes.

**Results:**

*I*_Na_ conductance density was ∼1.8-fold greater in atrial than in ventricular cells. Atrial *I*_Na_ was activated at command potentials ∼7 mV more negative and inactivated at conditioning potentials ∼11 mV more negative than ventricular *I*_Na_. The onset of inactivation of *I*_Na_ was faster in atrial cells than in ventricular myocytes. Ranolazine (30 μM) inhibited *I*_Na_ in atrial and ventricular myocytes in a use-dependent manner consistent with preferential activated/inactivated state block. Ranolazine caused a significantly greater negative shift in voltage of half-maximal inactivation in atrial cells than in ventricular cells, the recovery from inactivation of *I*_Na_ was slowed by ranolazine to a greater extent in atrial myocytes than in ventricular cells, and ranolazine produced an instantaneous block that showed marked voltage dependence in atrial cells.

**Conclusion:**

Differences exist between rabbit atrial and ventricular myocytes in the biophysical properties of *I*_Na_. The more negative voltage dependence of *I*_Na_ activation and inactivation, together with trapping of the drug in the inactivated channel, underlies an atrial-selective action of ranolazine.

## Introduction

Atrial fibrillation (AF), characterized by rapid and irregular electrical activation of the atria, reduced cardiac output, poor response to exercise, and fatigue, is the most commonly occurring clinical arrhythmia.^1^ AF is associated with significant morbidity and mortality, principally through an elevated risk of thromboembolism and ischemic stroke owing to inadequate emptying of the atria, although the elevated ventricular rate can also contribute to tachycardia-induced cardiomyopathy and decompensated heart failure.^1^ The condition tends to be progressive, with paroxysms of AF leading with time to persistent and permanent AF.^1^ The progressive nature of AF is thought to arise through the elevated rate causing electrical and structural remodeling that stabilizes the arrhythmia.^1^ Early intervention to prevent and/or control the arrhythmia is therefore highly desirable.^1^, ^2^

The activation of voltage-gated Na^+^ channels underlies the propagation and conduction of the action potential through the heart, whereas their subsequent inactivation initiates a refractory period that is usually determined by the duration of the action potential.^3^ Voltage-gated Na^+^ channels are a major target for antiarrhythmic drugs, as the combined effects of reduction in membrane excitability, conduction velocity slowing, and prolongation of the refractory period can both suppress triggered activity and extinguish reentrant activity.^2^ Blockers of voltage-gated Na^+^ channels with relatively slow dissociation kinetics (ie, the class Ia and Ic antiarrhythmic drugs of the Vaughan-Williams classification) are effective in the cardioversion of early-onset AF and the maintenance of sinus rhythm.^4^, ^5^ The class Ic drugs, flecainide and propafenone, are recommended as a suitable “pill-in-the-pocket.”^5^, ^6^

Despite the effectiveness of class Ic antiarrhythmic drugs in the treatment of AF, the Cardiac Arrhythmia Suppression Trial demonstrated that these drugs carried an increased mortality in patients with structural abnormalities, precluding their use in such patients.^7^ Consequently, there has been considerable interest in alternative agents that allow atrial-selective targeting of voltage-gated Na^+^ channels.^2^, ^8^, ^9^ In principle, atrial selectivity of action might arise through (1) atrial-ventricular differences in the molecular, biophysical, or pharmacologic properties of voltage-gated Na^+^ channels and/or (2) atrial-ventricular differences in the resting membrane potential and in configuration of the action potential. Evidence from canine, guinea pig, rabbit, and rat cardiac myocytes supports the existence of atrial-ventricular differences in Na^+^ channel function: compared with the voltage-gated Na^+^ channel current (*I*_Na_) of ventricular myocytes, atrial *I*_Na_ inactivates at more negative voltages, with more rapid onset, and recovers more slowly from inactivation.^10^, ^11^, ^12^, ^13^

The antianginal drug ranolazine (Ranexa) is a prototypic example of a drug with a putative atrial-selective action against voltage-gated Na^+^ channels.^8^, ^11^ Ranolazine has been found to suppress the incidence of AF in anginal patients and is suggested to be effective in pharmacologic cardioversion of patients with early-onset AF.^14^, ^15^, ^16^ Ranolazine shows structural homology to lidocaine and has been suggested to bind to the local anesthetic binding site within the central cavity of the pore.^17^, ^18^ However, the mechanism for the atrial-selective action of the drug remains unclear. Preferential binding of ranolazine to the inactivated state of the channel and slowing of recovery from inactivation have been suggested to underlie use-dependent block, so that atrial selectivity arises from the inactivation of *I*_Na_ at more negative voltages in atrial cells.^19^, ^20^ On the other hand, ranolazine has been suggested to be an open channel blocker that becomes trapped in the inactivated state.^21^, ^22^ The objective of this study was to investigate atrial-ventricular differences in the properties of *I*_Na_ and its block by ranolazine in rabbit cardiac myocytes.

## Methods

Detailed methods are available in Supplemental Information online.

### Rabbit cardiac myocyte isolation

Rabbit right ventricular and left atrial myocytes were isolated as described previously, using procedures approved by the University of Bristol Animal Welfare and Ethics Board in accordance with UK legislation and the *Guide for the Care and Use of Laboratory* Animals.^23^, ^24^

### Whole-cell recording of voltage-gated Na^+^ currents

Whole-cell Na^+^ currents (*I*_Na_) were recorded at room temperature using the patch-clamp technique and symmetrical internal and external [Na^+^] (10 mM).

### Ranolazine

Ranolazine (Sequoia Research Products Ltd, Pangbourne, UK) was used at 30 μM to produce significant use-dependent block of the fast component of *I*_Na_.^22^

### Statistics

Data are presented as the mean ± standard error of the mean. *P* < .05 was used as the limit of statistical confidence. Curve fitting was performed by nonlinear least squares using Igor Pro v6 (WaveMetrics Inc, Portland, OR).

## Results

**Figure 1:**
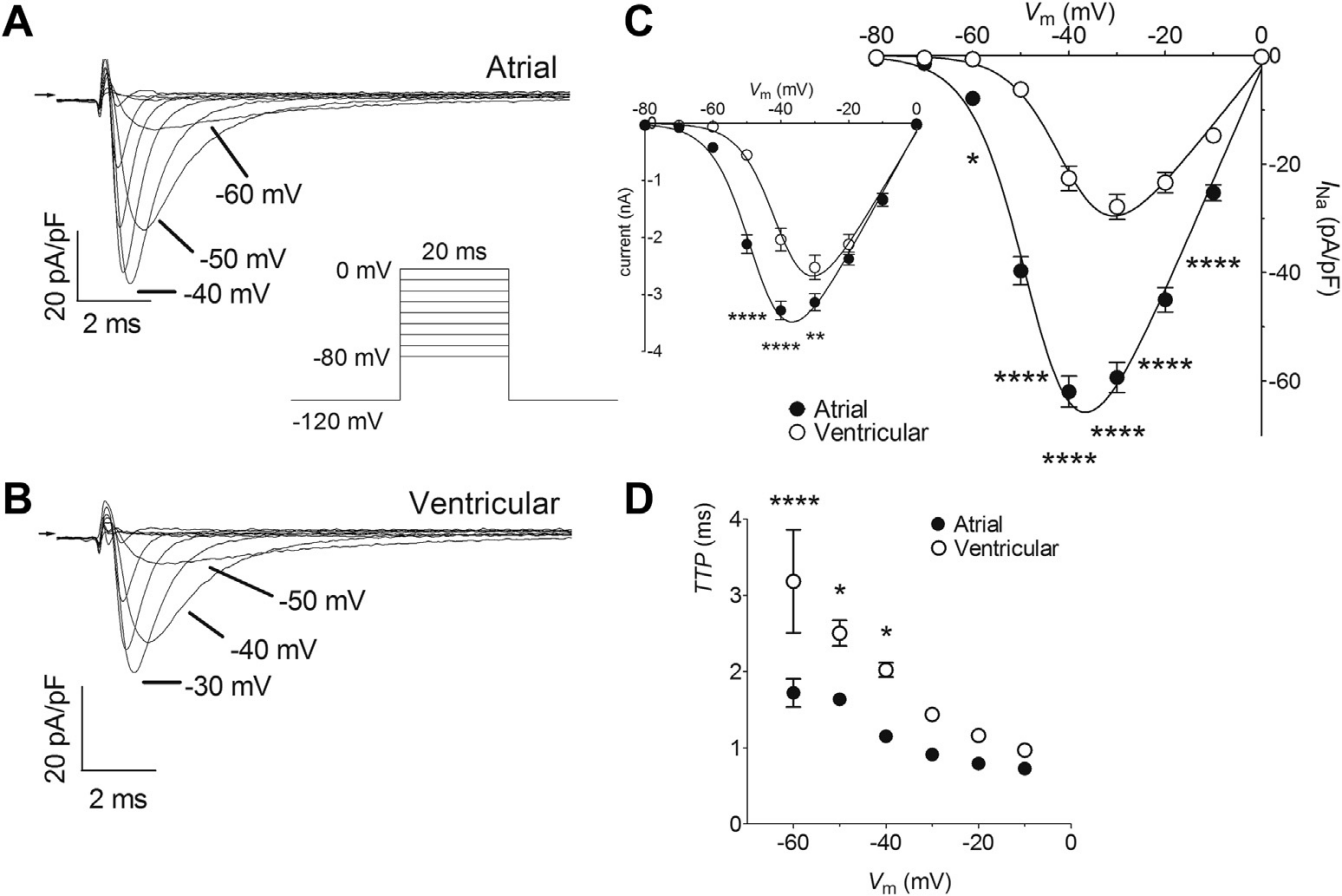
Atrial-ventricular differences in fast-Na^+^ current (*I*_Na_) density–voltage relations. **A:** Representative current traces recorded from an atrial myocyte on depolarization to a range of voltages. Arrow indicates zero current level. Insert shows voltage pulse protocol. **B:** Representative current traces recorded from a ventricular myocyte on depolarization to a range of voltages. Arrow indicates zero current level. Voltage pulse protocol as for A. **C:** Mean *I*_Na_ density–voltage relations for atrial (filled circles, n = 17) and ventricular (open circles, n = 17) myocytes. Solid lines represent fits to Supplemental Equation 1. Data were significantly different by both cell type (*P* < .0001) and voltage (*P* < .0001), with significant interaction (*P* < .0001; 2-way repeated measures analysis of variance [RM ANOVA]). *P < .05; ****P < .0001 vs ventricular; Bonferroni post hoc test. Inset shows the corresponding mean *I*_Na_ density–voltage relations without normalization to whole-cell capacitance. Data were significantly different by both cell type (*P* = .0002) and voltage (*P* < .0001), with significant interaction (*P* < .0001; 2-way RM ANOVA). **P < .01; ****P < .0001 vs ventricular; Bonferroni post hoc test. **D:** Voltage dependence of time-to-peak *I*_Na_ (TTP) for atrial (filled circles, n = 17) and ventricular (open circles, n = 17) myocytes. Data were significantly different by both cell type (*P* < .0001) and voltage (*P* < .0001), with significant interaction (*P* = .0355; 2-way RM ANOVA). *P < .05; ****P < .0001 vs ventricular; Bonferroni post hoc test. The membrane time constants were 0.168 ± 0.009 ms for atrial myocytes (n = 17) and 0.327 ± 0.026 ms for ventricular cells (n = 17; *P* < .0001, unpaired Student *t* test).

Depolarizing pulses activated inward currents with rapid kinetics of activation and inactivation typical of *I*_Na_ in both atrial and ventricular myocytes (Figure 1A and B). The currents of both cell types showed a U-shaped current density–voltage relation with a zero-current potential close to zero mV, consistent with their Na^+^ selectivity (Figure 1C). However, *I*_Na_ from atrial myocytes activated at more negative voltages than ventricular *I*_Na_, with measurable inward currents being evident from voltages of −60 mV and positive and reaching a maximum at approximately −40 mV in atrial cells, whereas *I*_Na_ in ventricular myocytes were activated from −50 mV and reached a maximum at ∼−30 mV (Figure 1C). The current density–voltage relations of each cell type were fitted by a modified Boltzmann relation (Supplemental Equation 1) and the more negative voltage dependence of activation of atrial *I*_Na_ was reflected in a mean half-maximal voltage of activation (*V*_*half,act*_) approximately 7 mV more negative than that of ventricular *I*_Na_ (*P* < .0001; Supplemental Table 1). Although there was no difference in the slope factors, atrial myocytes showed a maximal conductance density almost twice that of ventricular cells (*P* < .0001; Supplemental Table 1). Atrial *I*_Na_ also showed shorter times to peak current than ventricular currents, suggesting more rapid activation of atrial voltage-gated Na^+^ channels (Figure 1D).

**Figure 2:**
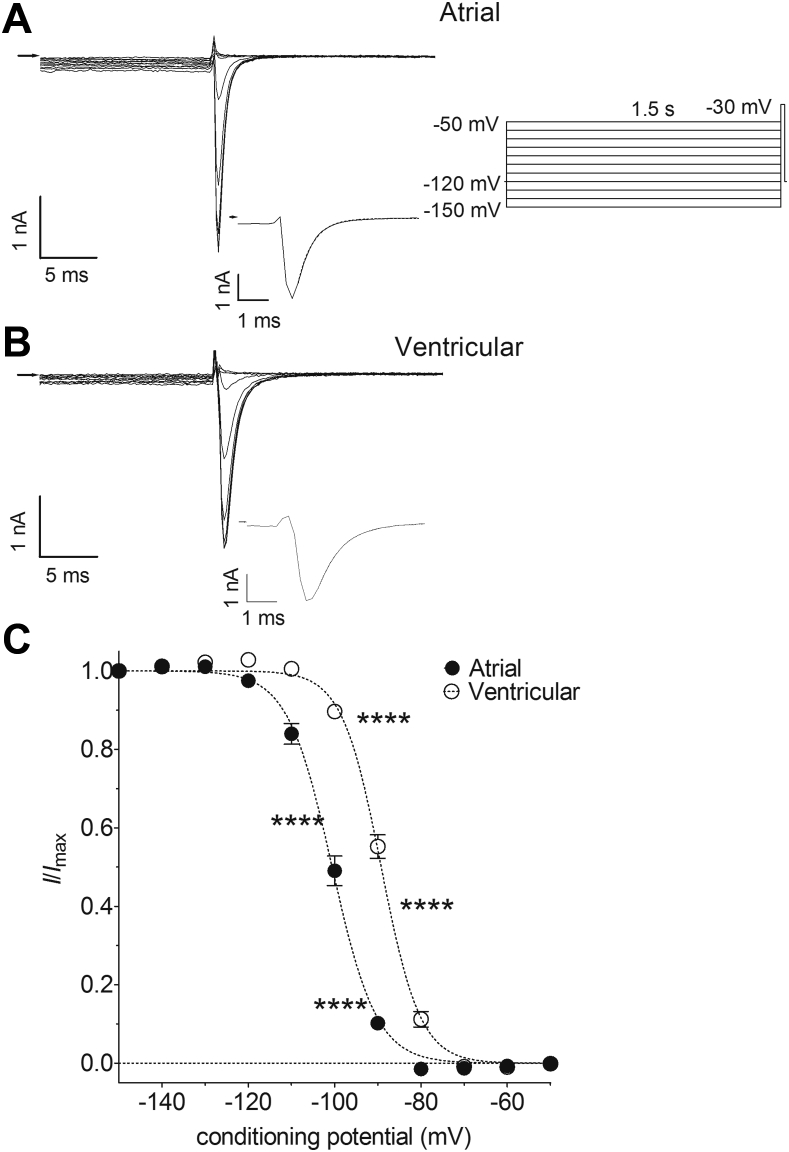
Atrial-ventricular differences in steady-state voltage-dependent inactivation of *I*_Na_. **A:** Representative current traces recorded from an atrial myocyte on depolarization to −30 mV after conditioning at a range of voltages. Arrow indicates zero current level. Inserts show voltage pulse protocol and current trace elicited from a conditioning potential of −150 mV on an expanded time scale. Dashed line represents fits to Supplemental Equation 2. **B:** Representative current traces recorded from a ventricular myocyte on depolarization to −30 mV after conditioning at a range of voltages. Arrow indicates zero current level. Voltage pulse protocol as in A. Insert shows current trace elicited from a conditioning potential of −150 mV on an expanded time scale. Dashed line represents fits to Supplemental Equation 2. **C:** Mean *I*_Na_ steady-state voltage-dependent inactivation curves for atrial (*filled circles*, n = 29) and ventricular (*open circles*, n = 29) myocytes. *I*_Na_ were normalized to the amplitude from a conditioning potential of −150 mV. Dashed lines represent fits to Supplemental Equation 3. Data were significantly different by both cell type (*P* < .0001) and voltage (*P* < .0001), with significant interaction (*P* < .0001; 2-way repeated measures analysis of variance). ****P < .0001 vs ventricular; Bonferroni post hoc test.

Both atrial and ventricular *I*_Na_ showed voltage-dependent inactivation in response to 1.5-second conditioning pulses from −150 mV to −50 mV (Figure 2). Currents were maximal from conditioning potentials of −130 mV and negative but showed voltage-dependent inactivation at more positive potentials. The rate of onset of *I*_Na_ inactivation was examined by fitting a single decaying exponential relation (Supplemental Equation 2) to the currents activated from a conditioning potential of −150 mV (see inserts to Figure 2A and B). Inactivation of *I*_Na_ was faster in atrial cells than in ventricular myocytes (τ: atrial, 0.64 ± 0.02 ms, n = 29; ventricular, 1.11 ± 0.03 ms, n = 29; *P* < .0001, unpaired Student *t* test). Atrial *I*_Na_ also inactivated at more negative voltages than the current in ventricular cells, with significant differences evident between the 2 cell types in the range from −110 mV to −80 mV (Figure 2C). The voltage dependence of inactivation was fitted by a Boltzmann relation (Supplemental Equation 3) and the mean voltage of half-maximal inactivation (*V*_*half,inact*_) of atrial cells was ∼11.5 mV more negative than that of ventricular myocytes (Supplemental Table 1).

**Figure 3:**
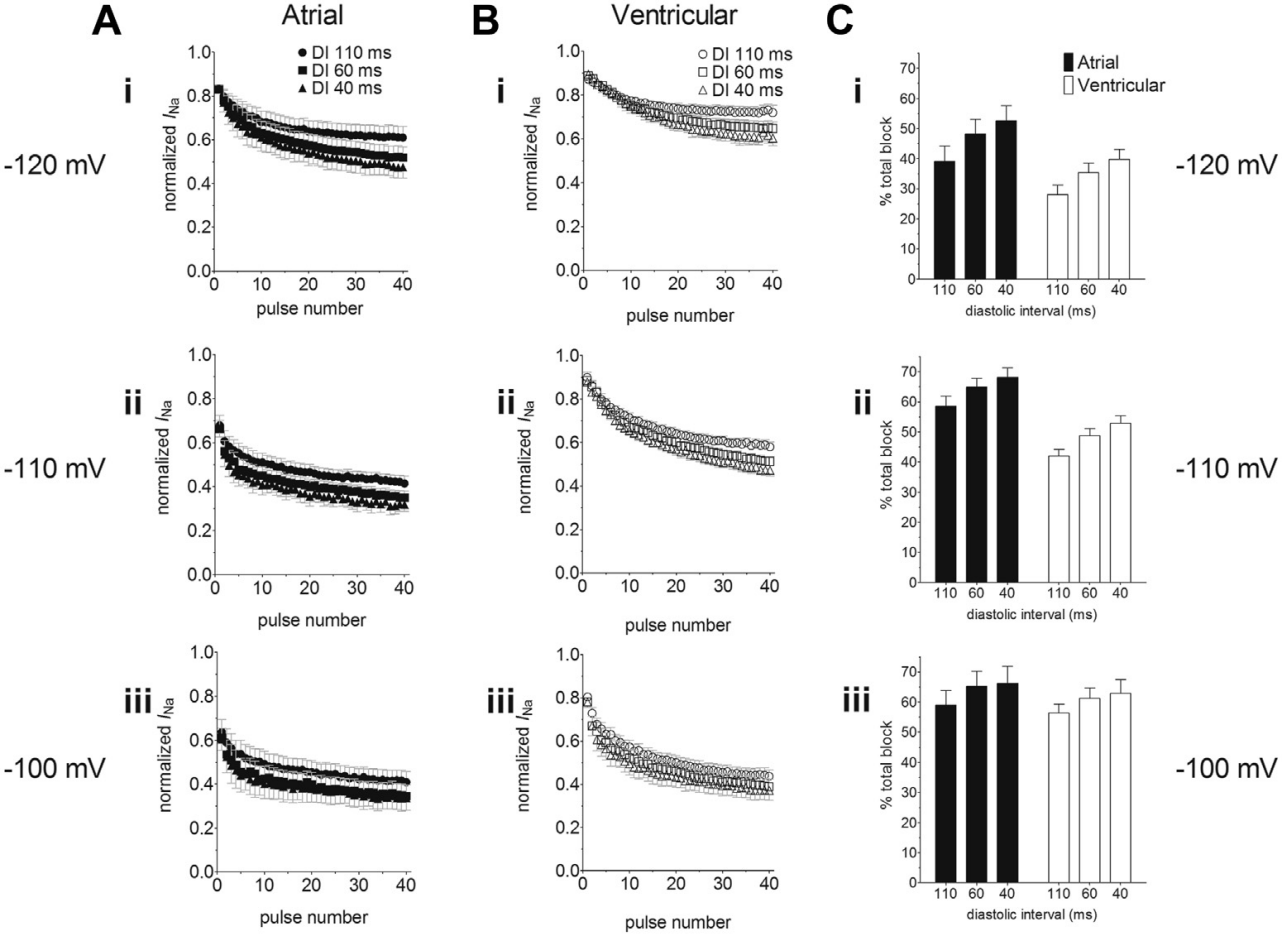
Use-dependent block of *I*_Na_ by ranolazine (RAN, 30 μM). **A:** Mean normalized current amplitudes recorded by a series of 40 pulses to −30 mV at diastolic interval (DI) of 110 ms (*circles*), 60 ms (*squares*), and 40 ms (*triangles*) in atrial myocytes (*filled symbols*) from holding potentials (HPs) of (i) −120 mV (n = 6), (ii) −110 mV (n = 6), and (iii) −100 mV (n = 5) in the presence of RAN. Currents were normalized to the currents elicited in the absence of RAN by the corresponding pulse number. **B:** Mean normalized current amplitudes recorded using the same protocol as used in A from ventricular myocytes (*open symbols*) in the presence of RAN from HPs of (i) −120 mV (n = 5), (ii) −110 mV (n = 6), and (iii) −100 mV (n = 9). **C:** The mean percentage total block elicited by the 40th pulse at DIs of 110, 60, and 40 ms from atrial (*filled columns*) and ventricular (*open columns*) myocytes at HPs of (i) −120 mV, (ii) −110 mV, and (iii) −100 mV (sample sizes correspond to A and B). Total block was significantly different by factorial mixed analysis of variance (*P* < .001). Data were significantly different by DI (*P* < .001), HP (*P* < .001), and cell type (*P* < .001). There was a significant interaction between cell type and HP (*P* = .02). In Bonferroni post hoc tests, the effect of 110 ms DI was significantly different from 60 ms (*P* = .014) and 40 ms (*P* < .001) but there was no statistical confidence in the difference between DI of 60 ms and 40 ms (*P* = .558). Similarly, the effect of an HP of −120 mV was significantly different from both −110 mV (*P* < .001) and −100 mV (*P* < .001) and the effect of −110 mV was significantly different from −100 mV (*P* = .046).

The use-dependent interaction of ranolazine (RAN, 30 μM) with voltage-gated Na^+^ channels in rabbit atrial and ventricular myocytes was investigated by examining the effects of shortening of diastolic interval (DI) and of changing the holding potential (HP) on the degree of block during fixed trains of 40 consecutive pulses of 20 ms duration to −30 mV (Figure 3). The effects of DI of 110 ms, 60 ms, and 40 ms were examined in each cell at each HP. If the drug interaction with the resting channel is weak, then RAN will tend to dissociate during the DI at an HP of −120 mV. Reduction of DI can be expected to lead to accumulation of block as the time for unbinding becomes abbreviated. The effect of voltage on block was examined by performing experiments at HPs of −120 mV, −110 mV, and −100 mV in different cells (respectively, panels i, ii, and iii in Figure 3A and B). Depolarization of the HP can be expected to increase accumulation of block, as recovery from inactivation to the rested state will be limited. As reported for currents through recombinant voltage-gated Na^+^ channel subunits and endogenous channels in canine cardiac myocytes,^20^, ^21^, ^22^, ^25^, ^26^, ^27^ 30 μM RAN inhibited *I*_Na_ in a use-dependent manner, the level of block accumulating over the 40 consecutive pulses, the block being greater at shorter DI (Figure 3A and B). A degree of instantaneous block (10%–40%) was evident at the first pulse and the degree of block increased over subsequent pulses, reaching between 25% and 70% total block at the 40th pulse. The total level of block achieved on the 40th pulse depended on DI (*P* < .001, factorial mixed analysis of variance [ANOVA]) and HP (*P* < .001; Figure 3C). The inhibition of *I*_Na_ by RAN was different between atrial and ventricular myocytes (*P* < .001) and there was an interaction between cell type and HP (*P* = .02). This is clear from panels i, ii, and iii of Figure 3C: in both atrial and ventricular cells, mean total block increased with shortening DI at each HP. However, whereas in ventricular cells total block at each DI increased with less negative HP over the range from −120 mV to −100 mV, in atrial cells the voltage dependence of block was apparent primarily between −120 mV and −110 mV. In consequence, the differences between atrial and ventricular cells in total block by RAN were most marked at an HP of −110 mV (Figure 3C). In summary, the data suggest a difference between atrial and ventricular myocytes in the voltage dependence of block by RAN.

**Figure 4:**
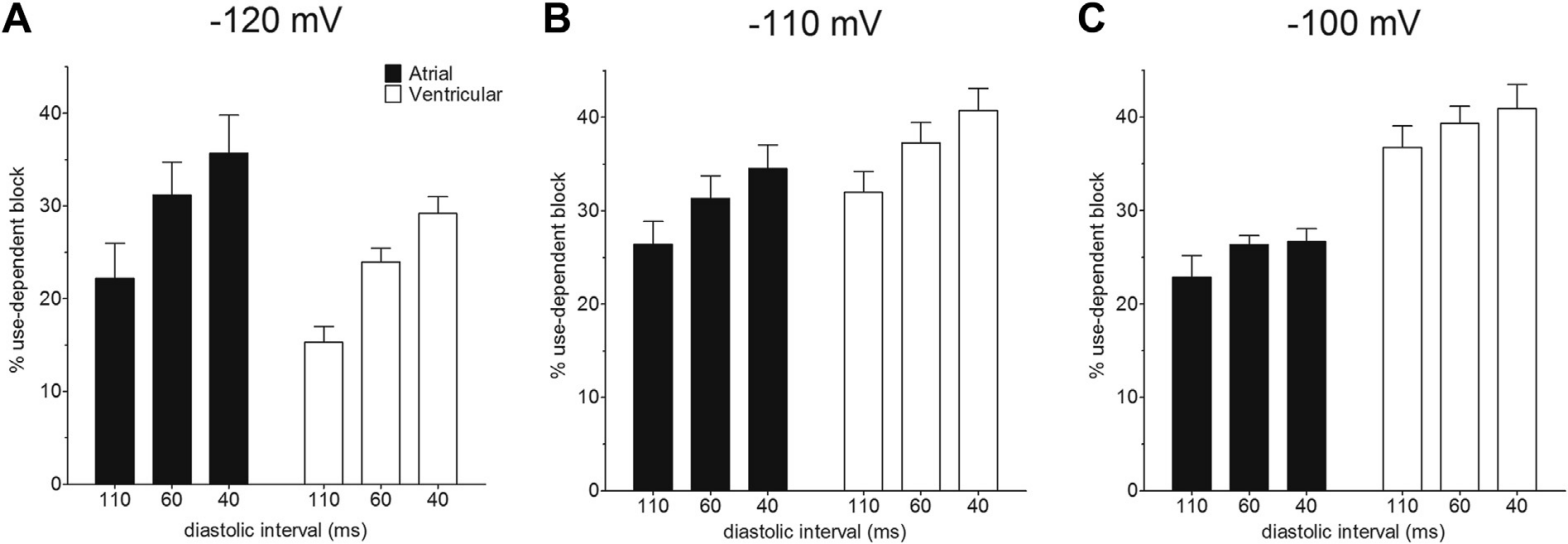
Effect of holding potential (HP) on percentage use-dependent block by ranolazine. **A:** Mean percentage use-dependent block at diastolic interval (DI) of 110, 60, and 40 ms in atrial (*filled columns*, n = 6) and ventricular (*open columns*, n = 5) from an HP of −120 mV. **B:** Percentage use-dependent block at DI of 110, 60, and 40 ms in atrial (*filled columns*, n = 6) and ventricular (*open columns*, n = 6) from an HP of −110 mV. **C:** Percentage use-dependent block at DI of 110, 60, and 40 ms in atrial (*filled columns*, n = 5) and ventricular (*open columns*, n = 9) from an HP of −100 mV. Use-dependent block was significantly different by factorial mixed analysis of variance (*P* < .001). Data were significantly different by DI (*P* < .001), HP (*P* < .001), and cell type (*P* = .001). There was a significant interaction between cell type and HP (*P* < .001). In Bonferroni post hoc tests, the effect of 110 ms DI was significantly different from 60 ms (*P* = .001) and 40 ms (*P* < .001) but there was no statistical confidence in the difference between DI of 60 ms and 40 ms (*P* = .146). Similarly, the effect of an HP of −120 mV was significantly different from both −110 mV (*P* < .001) and −100 mV (*P* < .001) but the effect of −110 mV was not different from −100 mV (*P* = 1.000).

**Figure 5:**
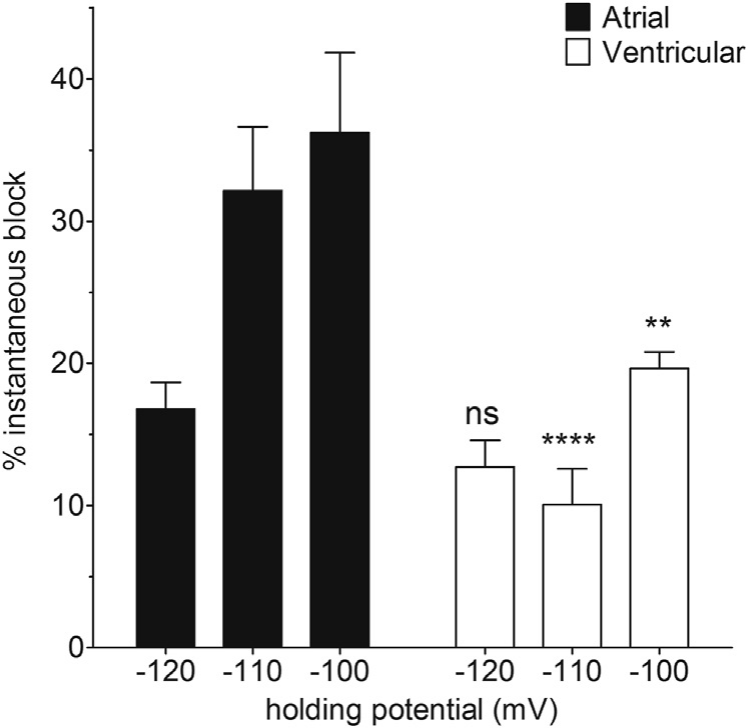
Effect of holding potential (HP) on instantaneous block by ranolazine. Data show mean percentage block on the first pulse to −30 mV at a diastolic interval of 110 ms from HPs of −120, −110, and −100 mV in atrial (*filled columns*; respectively, 6, 6, and 5 cells) and ventricular (*open columns*; respectively, 5, 6, and 9 cells) myocytes. **P < .01; ****P < .0001; 2-way analysis of variance with Bonferroni post hoc test vs atrial cells at the corresponding HP.

Use-dependent block by RAN, quantified as the difference in percentage block between the first and the 40th pulse, was also different between atrial and ventricular cells (factorial mixed ANOVA, *P* = .001; Figure 4). The percentage use-dependent block ranged from ∼15% to ∼40%. Disregarding cell type and HP, use-dependent block was increased at shorter DI (*P* < .001), consistent with preferential association of the drug with activated states of the channel. Similarly, disregarding cell type and DI, use-dependent block was different by HP (*P* < .001). However, though the increase in use-dependent block at shorter DI was similar between atrial and ventricular cells, the effect of HP differed between the 2 cell types: whereas, over the 3 DI, use-dependent block increased at more depolarized HPs in ventricular cells, use-dependent block was *reduced* by depolarization of the HP in atrial cells; this was particularly evident at the shorter DI. The difference between atrial and ventricular cells in the voltage dependence of use-dependent block is reflected in the significant interaction between cell type and HP for these data (*P* < .001; Figure 4). As a result, the use-dependent block at each DI was *smaller* in atrial cells than in ventricular myocytes at −100 mV (Figure 4C). There were also marked differences between the 2 cell types in the instantaneous block (*P* < .0001, 2-way ANOVA; Figure 5). Whereas in atrial cells instantaneous block was markedly increased by depolarization of the HP, there was no obvious voltage dependence to instantaneous block in ventricular cells. In consequence, instantaneous block was significantly greater in atrial cells compared with ventricular cells at −110 mV and −100 mV (Figure 5).

**Figure 6:**
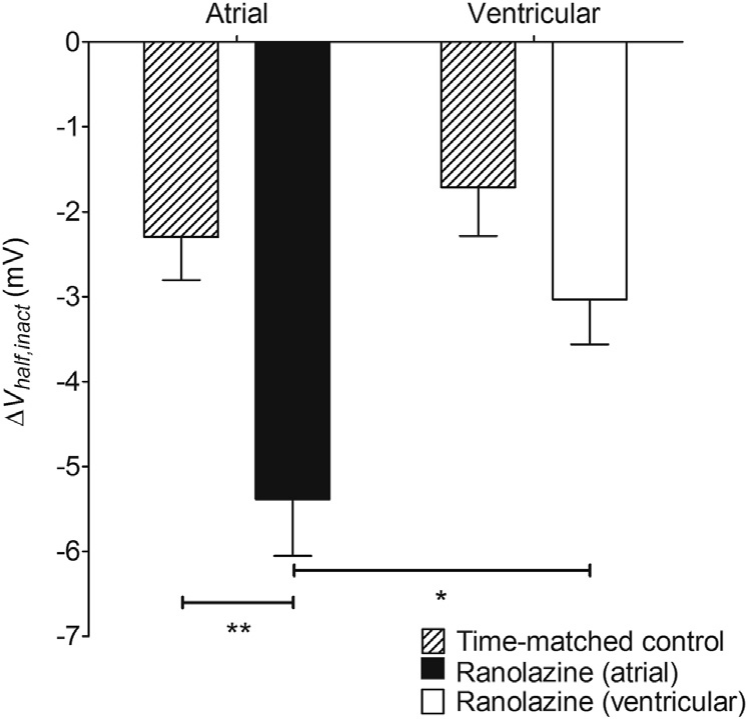
Effect of ranolazine (RAN) on half-maximal voltage of steady-state inactivation. Data shown are the mean changes in *V*_half,inact_ caused by 30 μM RAN for atrial (*filled column*, n = 7) and ventricular (*open column*, n = 5) myocytes. Hatched columns show corresponding time-matched controls in the absence of RAN for 6 atrial and 5 ventricular myocytes. *P < .05; **P < .01; 2-way analysis of variance with Bonferroni post hoc test.

The effect of RAN on steady-state inactivation was examined in atrial and ventricular myocytes. In both cell types, treatment with RAN was associated with a negative shift in *V*_*half,inact*_ (Figure 6). The shift was approximately 1.8-fold greater in atrial myocytes than in ventricular myocytes (*P* < .05). However, time-matched control experiments showed a time-dependent shift in *V*_*half,inact*_ in atrial and ventricular myocytes, with no significant difference between the cell types in the magnitude of the shift (Figure 6). Although the RAN-induced shift in *V*_*half,inact*_ was significantly greater than the time-matched control in atrial myocytes (*P* < .01), this was not the case in ventricular cells. Thus, RAN caused a negative shift in *V*_*half,inact*_ that achieved the level of statistical confidence in atrial myocytes. RAN also caused an apparent acceleration in the time constant of inactivation from a conditioning potential of −150 mV in both atrial (12.4% ± 2.23%, n = 7, *P* = .0015, paired *t* test) and ventricular (11.7% ± 1.72%, n = 5, *P* = .0024, paired *t* test) myocytes. There was no difference between the 2 cell types in the acceleration of inactivation (P = .8429, unpaired *t* test).

**Figure 7:**
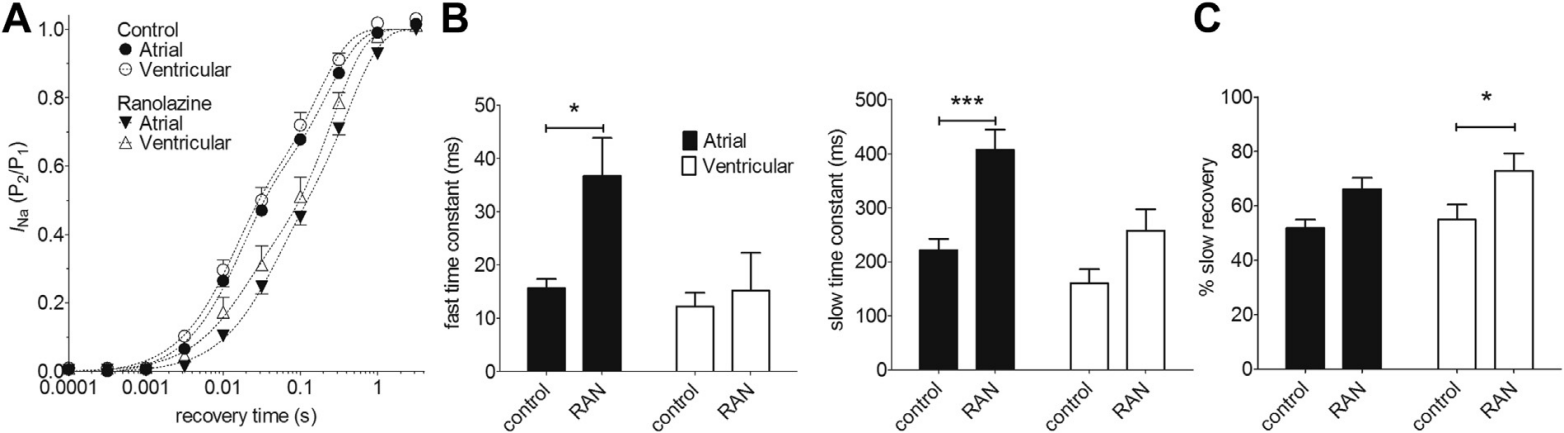
Effect of ranolazine (RAN) on *I*_Na_ recovery from inactivation. **A:** Recovery of *I*_Na_ from inactivation in atrial (*filled symbols*, n = 6) and ventricular (*open symbols*, n = 5) myocytes. Dashed lines represent fits to Supplemental Equation 4. The holding potential during recovery was −120 mV. **B:** Fitted fast (left-hand panel) and slow (right-hand panel) time constants in control and in the presence of 30 μM RAN for atrial (*filled columns*) and ventricular (*open columns*) myocytes. *P < .05; ***P < .001; 2-way repeated measures analysis of variance (RM ANOVA) with Bonferroni post hoc test vs control. **C:** Mean amplitude of slow component in control and in the presence of 30 μM RAN for atrial (*filled columns*) and ventricular (*open columns*) myocytes. *P < .05; 2-way RM ANOVA with Bonferroni post hoc test vs control.

In both atrial and ventricular myocytes, *I*_Na_ recovered from inactivation with a biexponential time course, with a fast time constant (*τ*_*f*_) of 10–20 ms and a slow time constant (*τ*_*s*_) of 120–300 ms (Figure 7A). The fast and slow components contributed approximately equally to recovery in both cell types, and although the mean fast and slow time constants were larger (ie, slower) in atrial than in ventricular cells, this did not achieve the level of statistical confidence (Figure 7B and C). The recovery from inactivation was slowed by RAN in both cell types. However, RAN appeared more effective in atrial cells, slowing mean *τ*_*f*_ by 2.4-fold (*P* < .05) and *τ*_*s*_ by 1.8-fold (*P* < .001), whereas in ventricular cells mean *τ*_*f*_ was slowed by 1.5-fold (not statistically significant) and *τ*_*s*_ by 1.6-fold (*P* < .05) (Figure 7B). The contribution of the slow component of recovery was increased by RAN by 15%–20% in both cell types (Figure 7C).

## Discussion

### Atrial-ventricular differences in *I*_Na_

Clear atrial-ventricular differences were found in the biophysical properties of rabbit cardiac voltage-gated Na^+^ currents: atrial *I*_Na_ was activated at command potentials ∼7 mV more negative and inactivated at conditioning potentials ∼11 mV more negative than ventricular *I*_Na_, and the onset of activation and inactivation of *I*_Na_ were also faster in atrial cells than in ventricular myocytes. In these respects, the differences in *I*_Na_ between rabbit atrial and ventricular cells in the present study were similar to those reported previously in cardiac myocytes from various species.^10^, ^11^, ^12^, ^13^ On the other hand, in contrast to previous reports from rat and guinea pig cardiac myocytes, there was no significant difference between rabbit atrial and ventricular cells in the rate of recovery of *I*_Na_ from inactivation in the present study.^10^, ^12^ Significant atrial-ventricular differences in block of *I*_Na_ by ranolazine were also evident: (1) ranolazine caused a significantly greater negative shift in *V*_*half,inact*_ in atrial cells than in ventricular cells, as has been reported previously in canine cardiac myocytes^11^; (2) the recovery from inactivation of *I*_Na_ was slowed by ranolazine to a greater extent in atrial myocytes than in ventricular cells; and (3) although there was little effect of HP on instantaneous block in ventricular cells, in atrial cells ranolazine produced an instantaneous block that showed marked voltage dependence, increasing from ∼16% block at an HP of −120 mV to ∼36% at −100 mV. The atrial-selective slowing of recovery from inactivation contrasts somewhat with a report showing little atrial-ventricular difference in the recovery from block in canine cardiac myocytes.^22^ Although voltage-dependent instantaneous block by ranolazine has been reported to be present in canine atrial cells and absent in canine ventricular cells, the degree of voltage dependence and the level of instantaneous block achieved in that study (ie, mean block ∼4% at −120 mV and 6% at −100 mV) were much smaller than those found in rabbit atrial myocytes in the present study.^22^ Though atrial-ventricular differences in the effects of ranolazine on the late Na^+^ current have previously been reported in rabbit cardiac myocytes, to the best of our knowledge this represents the first report of atrial-ventricular differences in the effects of ranolazine on the fast component of *I*_Na_ in rabbit cells.^28^

### Mechanism of ranolazine block

**Figure 8:**
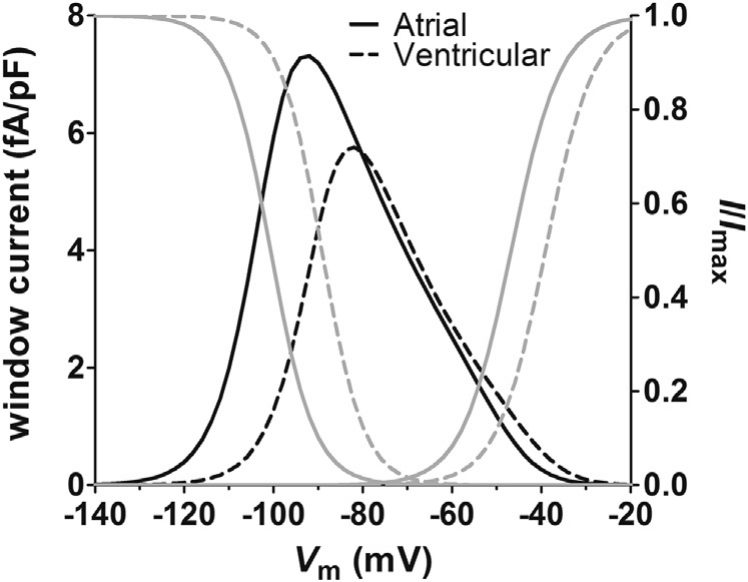
The window current in atrial and ventricular myocytes. Data plotted against the left-hand axis are window current densities calculated from the parameters presented in Supplemental Table 1 for atrial (*solid black line*) and ventricular (*dashed black line*) myocytes. Data plotted against the right-hand axis show steady-state voltage-dependent activation and inactivation curves (*I*/*I*_max_) according to the fitted parameters presented in Supplemental Table 1 for atrial (*solid gray lines*) and ventricular (*dashed gray lines*) myocytes.

The data are consistent with preferential interaction of ranolazine with activated states of both atrial and ventricular Na^+^ channels, as has been suggested previously.^22^, ^29^ Although we did not investigate the possibility of ranolazine interaction with the inactivated state of the channel (eg, by varying the duration of the test pulses in the trains), consideration of the physicochemical properties of the drug and the structure of voltage-gated Na^+^ channels indicates that preferential binding to the inactivated state is unlikely: Ranolazine is thought to interact with the local anesthetic binding site of voltage-gated Na^+^ channels.^17^, ^21^ As ranolazine has pKa values of 2.8 and 7.2, approximately 50% of the drug will be charged at physiological pH.^30^ Access of the drug to the binding site is therefore most likely through the cytosolic mouth to the pore and would require channel activation. However, the degree of instantaneous block by ranolazine evident at negative HPs at which there was very little *I*_Na_ activation in the present study is striking, particularly in atrial myocytes (Figure 1). Although fenestrations identified in the x-ray crystallographic structure of Na^+^ channels have been suggested to represent hydrophobic pathways for access of neutral drugs to the local anesthetic binding site, the relatively large size of ranolazine (molecular weight ∼427.5 g.mol^−1^, ∼22 Å length) argues against significant access of the drug via that route.^29^, ^31^, ^32^ On the other hand, overlap in the voltage dependence of steady-state activation and inactivation will result in a small, but nevertheless significant, window current at negative voltages in both atrial and ventricular myocytes (Figure 8). Thus, the instantaneous block likely results from the small proportion of channels activated at negative HPs. Notably, the voltage dependence of instantaneous block in atrial myocytes corresponded with the current density–voltage relation of the window current. Thus, the difference between atrial and ventricular cells in the voltage dependence of instantaneous block likely reflects differences in the voltage dependence of activation of the window current in the 2 cell types. Similarly, the greater degree of instantaneous block in rabbit atrial myocytes in the present study as compared with canine atrial myocytes presumably reflects the more negative position of the window current on the voltage axis relative to the previously published data.^11^, ^22^, ^29^ The mechanism underlying the more negative voltage dependence of *I*_Na_ activation and inactivation in the present study compared with previous reports is unclear but, in principle, could reflect differences in recording conditions (eg, temperature, composition of solutions) and/or species differences in the biophysical properties of cardiac Na^+^ channel isoforms.^10^, ^11^, ^12^, ^13^, ^22^, ^29^

The accentuation of use-dependent block by ranolazine at shorter DIs in atrial and ventricular myocytes in the present study reflected incomplete recovery from block during the DI when the channels tend to return toward the resting state. The increase in use-dependent block with depolarized HP in ventricular myocytes arises from slower recovery from activated/inactivated states to the resting state and slower dissociation of the drug at the more positive potentials as the drug is trapped in the inactivated channel. On the other hand, it was striking that the use-dependent block in atrial myocytes was *reduced* at depolarized HPs. This was presumably a consequence of the voltage dependence of instantaneous block in these cells: use-dependent block was reduced at depolarized potentials because the drug had gained access to the local anesthetic binding site via activation of the window current in these cells. Thus, the atrial-selective actions of ranolazine in the present study are largely consequent upon the more negative voltage dependence of activation and inactivation of *I*_Na_ in atrial cells as compared with ventricular myocytes.

## Conclusion

Differences exist between atrial and ventricular myocytes in the biophysical properties of *I*_Na_ in rabbit cardiac myocytes. The more negative voltage dependence of *I*_Na_ activation and inactivation, together with trapping of the drug in the inactivated channel, underlies atrial-selective *I*_Na_ inhibition by ranolazine.

## References

[1] Benjamin E.J., Chen P.-S., Bild D.E. (2009). Prevention of atrial fibrillation: report from a national heart, lung, and blood institute workshop. Circulation.

[2] Workman A.J., Smith G.L., Rankin A.C. (2011). Mechanisms of termination and prevention of atrial fibrillation by drug therapy. Pharmacol Ther.

[3] Carmeliet E., Vereecke J. (2002). Cardiac cellular electrophysiology.

[4] Lafuente-Lafuente C., Valembois L., Bergmann J.-F., Belmin J. (2015). Antiarrhythmics for maintaining sinus rhythm after cardioversion of atrial fibrillation. Cochrane Database Syst Rev.

[5] Camm A.J., Kirchhof P., Lip G.Y.H. (2010). Guidelines for the management of atrial fibrillation: The Task Force for the Management of Atrial Fibrillation of the European Society of Cardiology (ESC). Eur Heart J.

[6] Alboni P., Botto G.L., Baldi N., Luzi M., Russo V., Gianfranchi L., Marchi P., Calzolari M., Solano A., Baroffio R., Gaggioli G. (2004). Outpatient treatment of recent-onset atrial fibrillation with the “pill-in-the-pocket” approach. N Engl J Med.

[7] Echt D.S., Liebson P.R., Mitchell L.B. (1991). Mortality and morbidity in patients receiving encainide, flecainide, or placebo—the Cardiac Arrhythmia Suppression Trial. N Engl J Med.

[8] Burashnikov A., di Diego J.M., Zygmunt A.C., Belardinelli L., Antzelevitch C. (2008). Atrial-selective sodium channel block as a strategy for suppression of atrial fibrillation. Ann N Y Acad Sci.

[9] Hancox J.C., James A.F., Marrion N.V., Zhang H., Thomas D. (2016). Novel ion channel targets in atrial fibrillation. Expert Opin Ther Targets.

[10] Li G.-R., Lau C.-P., Shrier A. (2002). Heterogeneity of sodium current in atrial vs epicardial ventricular myocytes of adult guinea pig hearts. J Mol Cell Cardiol.

[11] Burashnikov A., Di Diego J.M., Zygmunt A.C., Belardinelli L., Antzelevitch C. (2007). Atrium-selective sodium channel block as a strategy for suppression of atrial fibrillation: differences in sodium channel inactivation between atria and ventricles and the role of ranolazine. Circulation.

[12] Chen K.-H., Xu X.-H., Sun H.-Y., Du X.-L., Liu H., Yang L., Xiao G.-S., Wang Y., Jin M.-W., Li G.-R. (2016). Distinctive property and pharmacology of voltage-gated sodium current in rat atrial vs ventricular myocytes. Heart Rhythm.

[13] Suzuki T., Morishima M., Kato S., Ueda N., Honjo H., Kamiya K. (2013). Atrial selectivity in Na^+^ channel blockade by acute amiodarone. Cardiovasc Res.

[14] Murdock D.K., Kersten M., Kaliebe J., Larrain G. (2009). The use of oral ranolazine to convert new or paroxysmal atrial fibrillation: a review of experience with implications for possible “pill in the pocket” approach to atrial fibrillation. Indian Pacing Electrophysiol J.

[15] Murdock D.K., Kaliebe J., Larrain G. (2012). The Use of ranolazine to facilitate electrical cardioversion in cardioversion-resistant patients: a case series. Pacing Clin Electrophysiol.

[16] Scirica B.M., Morrow D.A., Hod H., Murphy S.A., Belardinelli L., Hedgepeth C.M., Molhoek P., Verheugt F.W.A., Gersh B.J., McCabe C.H., Braunwald E. (2007). Effect of ranolazine, an antianginal agent with novel electrophysiological properties, on the incidence of arrhythmias in patients with non–ST-segment–elevation acute coronary syndrome: results from the Metabolic Efficiency with Ranolazine for Less Ischemia in Non–ST-Elevation Acute Coronary Syndrome–Thrombolysis in Myocardial Infarction 36 (MERLIN-TIMI 36) randomized controlled trial. Circulation.

[17] Fredj S., Sampson K.J., Liu H., Kass R.S. (2006). Molecular basis of ranolazine block of LQT-3 mutant sodium channels: evidence for site of action. Br J Pharmacol.

[18] Catterall W.A. (2012). Voltage-gated sodium channels at 60: structure, function and pathophysiology. J Physiol.

[19] Undrovinas A.I., Belardinelli L., Undrovinas N.A., Sabbah H.N. (2006). Ranolazine improves abnormal repolarization and contraction in left ventricular myocytes of dogs with heart failure by inhibiting late sodium current. J Cardiovasc Electrophysiol.

[20] Rajamani S., El-Bizri N., Shryock J.C., Makielski J.C., Belardinelli L. (2009). Use-dependent block of cardiac late Na^+^ current by ranolazine. Heart Rhythm.

[21] Wang G.K., Calderon J., Wang S.-Y. (2008). State- and use-dependent block of muscle Nav1.4 and neuronal Nav1.7 voltage-gated Na^+^ channel isoforms by ranolazine. Mol Pharmacol.

[22] Zygmunt A.C., Nesterenko V.V., Rajamani S., Hu D., Barajas-Martinez H., Belardinelli L., Antzelevitch C. (2011). Mechanisms of atrial-selective block of Na^+^ channels by ranolazine: I. Experimental analysis of the use-dependent block. Am J Physiol Heart Circ Physiol.

[23] Hancox J.C., Levi A.J., Lee C.O., Heap P. (1993). A method for isolating rabbit atrioventricular node myocytes which retain normal morphology and function. Am J Physiol.

[24] Garger J.C., Barbee R.W., Bielitzki J.T. (2011). Guide for the Care and Use of Laboratory Animals.

[25] El-Bizri N., Kahlig K.M., Shyrock J.C., George A.L., Belardinelli L., Rajamani S. (2011). Ranolazine block of human Nav1.4 sodium channels and paramyotonia congenita mutants. Channels (Austin).

[26] Peters C.H., Sokolov S., Rajamani S., Ruben P.C. (2013). Effects of the antianginal drug, ranolazine, on the brain sodium channel Nav1.2 and its modulation by extracellular protons. Br J Pharmacol.

[27] Rajamani S., Shryock J.C., Belardinelli L. (2008). Block of tetrodotoxin-sensitive, Nav1.7, and tetrodotoxin-resistant, Nav1.8, Na^+^ channels by ranolazine. Channels (Austin).

[28] Luo A., Ma J., Song Y., Qian C., Wu Y., Zhang P., Wang L., Fu C., Cao Z., Shryock J.C. (2014). Larger late sodium current density as well as greater sensitivities to ATX II and ranolazine in rabbit left atrial than left ventricular myocytes. Am J Physiol Heart Circ Physiol.

[29] Nesterenko V.V., Zygmunt A.C., Rajamani S., Belardinelli L., Antzelevitch C. (2011). Mechanisms of atrial-selective block of Na+ channels by ranolazine: II. Insights from a mathematical model. Am J Physiol Heart Circ Physiol.

[30] Wyatt K.M., Skene C., Veitch K., Hue L., McCormack J.G. (1995). The antianginal agent ranolazine is a weak inhibitor of the respiratory Complex I, but with greater potency in broken or uncoupled than in coupled mitochondria. Biochem Pharmacol.

[31] Payandeh J., Scheuer T., Zheng N., Catterall W.A. (2011). The crystal structure of a voltage-gated sodium channel. Nature.

[32] Martin L.J., Corry B. (2014). Locating the route of entry and binding sites of benzocaine and phenytoin in a bacterial voltage gated sodium channel. PLoS Comput Biol.

