# Supplementary material for: Atrial-ventricular differences in rabbit cardiac voltage-gated Na+ currents: Basis for atrial-selective block by ranolazine
Source: Heart Rhythm. 2017 Nov;14(11):1657–64. doi: 10.1016/j.hrthm.2017.06.012 (PMC5666337; doi:10.1016/j.hrthm.2017.06.012)
Supplement: Supplemental Information [file mmc1.docx]

**Supplemental Methods**

***Rabbit cardiac myocyte isolation***

Male New Zealand White rabbits (2.3±0.03 kg) were killed humanely in accordance with UK Home Office legislation using methods approved by the *Animal Welfare and Ethics Review Board* of the University of Bristol. Right ventricular and left atrial myocytes were isolated from 39 rabbits by enzymatic and mechanical dispersion as described previously^1^. Cells were stored in Kraftbrühe (KB) solution at 4 °C until use^2^.

***Whole-cell recording of voltage-gated Na^+^ currents***

Cells were placed in an experimental chamber mounted on the stage of an inverted microscope (Diaphot 200, Nikon UK Ltd, UK) and superfused with a Tyrode’s solution containing (in mM) NaCl 140, KCl 4, CaCl_2_ 1.2, MgCl_2_ 1, HEPES 5 and Glucose 10 at room temperature (pH 7.35 with NaOH). Whole-cell patch-clamp recordings were made using an EPC-9 amplifier (HEKA GmbH, Germany). Patch-pipettes (A-M Systems, USA) were pulled to a final resistance of 1-2 MΩ (P-97 Flaming/Brown Puller, Sutter Instruments, USA). Protocols were generated and data recorded on-line with Pulse software (v8.11, HEKA GmbH, Germany). Currents were subject to two low-pass 4-pole Bessel filters (*f*_c1_=10 kHz, *f*_c2_=2.9 kHz). Sampling frequencies were typically 10 – 100 kHz unless otherwise specified. Membrane currents were recorded in whole-cell voltage-clamp mode using an intracellular pipette solution containing (in mM) CsCl 130, NaCl 10, MgCl_2_ 5, Mg-ATP 5, HEPES 10, Glucose 5, BAPTA 5 (pH 7.2 with CsOH). The junction potential was compensated electronically on immersion of the pipette tip in the bath solution and no further compensation was applied. Voltage-gated Na^+^ currents (*I*_Na_) were recorded at room temperature from cells superfused with a low external [Na^+^] recording solution containing (in mM) CsCl 130, NaCl 10, CaCl_2_ 1.2, MgCl_2_ 1, HEPES 20, Glucose 11, nifedipine 0.02 (pH 7.35 with CsOH). Mean whole-cell capacitances were: atrial myocytes, 55±1.7 pF (*n*=53) and ventricular myocytes, 98±2.4 pF (*n*=52). Mean series resistances were: atrial, 3.7±0.2 MΩ and ventricular, 3.7±0.2 MΩ. Series resistance compensation was applied and was typically 50 – 60 %. No further correction was made for voltage-drop error, which was estimated to be between -4 to -5 mV at the maximal inward currents in both cell types.

***Voltage command protocols and data analysis***

*I*_Na_-voltage relations were obtained from atrial and ventricular myocytes by step depolarizations (20 ms duration, 1/5 s) to voltages of -80 mV to 0 mV from a holding potential of -120 mV. *I*_Na_ was measured as the difference between the peak inward current and the current at the end of the pulse. Currents were normalized to whole-cell capacitance as an index of cell surface area and expressed in pA/pF. Mean *I*_Na_ densities were plotted against the corresponding command voltage (*V_m_*) and fitted with a modified Boltzmann equation^3^:

*Equation 1*. $I_{Na}\left( V_{m} \right)=\frac{G_{max}.\left( V_{m}-V_{rev} \right)}{\left( 1+\left( \frac{\left( V_{m}-V_{half,act} \right)}{k} \right) \right)}$ ,

where *G_max_* represents the maximal Na^+^ conductance, *V_rev_* is the effective reversal potential, *V_half,act_* is the voltage of half-maximal current activation and *k* is a slope factor.

Steady-state voltage-dependent inactivation was examined by application of 1.5 s conditioning commands from -150 mV to -50 mV prior to activation of *I*_Na_ by a 20 ms depolarizing pulse to -30 mV (1/10 s). Currents were sampled at 5 kHz. The time course of *I*_Na_ inactivation following the peak inward current was fitted with a decaying exponential as follows:

*Equation 2*. $I_{Na}\left( t \right)=A.\exp\left( -\frac{t}{\tau} \right)+c$ ,

where *τ* is the time constant of inactivation and *c* is a non-inactivating leak current. *I*_Na_, measured as the difference between the peak inward current and the current at the end of the pulse, was normalized to the current from a conditioning potential of -150 mV (*I*_max_) and plotted against the corresponding conditioning potential. Steady-state inactivation curves were fitted with a Boltzmann relation, as follows:

*Equation 3*. $\frac{I}{I_{max}}(V_{m})= \frac{1}{(1+(\frac{(V_{half,inact}-V_{m})}{k}))}$ ‘

where *V_half,inact_* is the voltage of half-maximal inactivation and *k* is a slope factor.

Use-dependent block by ranolazine (RAN) was examined using a protocol developed from a previous report^4^ consisting of a consecutive series of 40 depolarizing pulses to -30 mV (20 ms) applied at diastolic intervals (DI) of 110, 60 and 40 ms. Currents were obtained by 40 consecutive pulses in control solution and then superfused with 30 μM RAN for ~30 s without stimulation before repeating the protocol in the continued presence of RAN. Currents in the presence of RAN were normalized to the current obtained by the corresponding pulse number in the absence of RAN and plotted against the pulse number. The effect of voltage on block by RAN was investigated by use of different holding potentials (-120, -110 and -100 mV) in different cells.

To examine the recovery of *I*_Na_ from inactivation, currents were activated and inactivated by a 1 s pulse to -30 mV from a holding potential -120 mV followed by a second pulse to -30 mV (10 ms) with a delay that varied from 0.1 to 3162 ms in half-log intervals. Currents were normalized to the current activated by the first pulse (*I_control_*) and plotted against the interval time. Data were fitted with the following equation.

*Equation 4*. $\frac{I}{I_{control}}\left( t \right)= 1-(A_{f}.\exp\left( \frac{-t}{\tau_{f}} \right)+A_{s}.\exp\left( \frac{-t}{\tau_{s}} \right))$ ,

where *τ_f_* and *τ_s_* are, respectively, the fast and slow time constants of recovery from inactivation and *A_f_* and *A_s_* are the corresponding amplitudes of the fast and slow components of recovery.

***Ranolazine***

Ranolazine (Sequoia Research Products Ltd, Pangbourne, UK) was made as a stock solution in de-ionized water, stored at -20 °C and diluted to the final concentration (30 μM) in low Na^+^ external recording solution on the day of experiment. The final concentration was chosen to produce significant use-dependent block of the fast component of *I*_Na_^4^.

***Statistics***

Data are presented as the mean ± standard error of the mean. Unless specified, comparisons between atrial and ventricular myocytes were made by Student’s unpaired t-test or two-way ANOVA with Bonferroni *post hoc* tests using Prism v5 (GraphPad Software Inc, CA, USA). Current density-voltage relations and the voltage-dependence of steady-state inactivation were compared by two-way repeated measures (RM) ANOVA and Bonferroni *post hoc* tests, with voltage as the repeated measure using Prism v5. The total and the use-dependent block by RAN in the two cell types were analyzed by factorial mixed ANOVA with Bonferroni *post hoc* tests using SPSS v23 (IBM UK Ltd., Portsmouth, UK). The instantaneous block by RAN was analyzed by two-way ANOVA with Bonferroni *post hoc* tests using Prism v5. P<0.05 was used as the limit of statistical confidence. Curve fitting was performed by non-linear least squares using Igor Pro v6 (Wavemetrics Inc, OR, USA).

**Supplemental Tables**

|  | Atrial | Ventricular |
| --- | --- | --- |
| **Activation** |  |  |
| Number of myocytes | 17 | 17 |
| *G_max_* (nS/pF) | 2.1 ± 0.1 | 1.1 ± 0.1**** |
| *V_half,act_* (mV) | -46.5 ± 0.7 | -39.3 ± 1.1**** |
| slope (mV) | 5.7 ± 0.4 | 5.4 ± 0.6 |
|  |  |  |
| **Inactivation** |  |  |
| Number of myocytes | 29 | 29 |
| *V_half,inact_* (mV) | -100.6 ± 0.3 | -89.2 ± 0.2**** |
| slope (mV) | -5.2 ± 0.2 | -4.5 ± 0.2* |

**Supplemental Table 1.** Fitted parameters of steady-state activation and inactivation to atrial and ventricular myocytes. Data presented in Figure 1 and Figure 2 were fitted with *Supplemental Equations 1* and *3*, respectively. *, P<0.05; ****, P<0.0001; Student’s unpaired t-test.

**References**

**1.** Hancox JC, Levi AJ, Lee CO, Heap P. A method for isolating rabbit atrioventricular node myocytes which retain normal morphology and function. Am J Physiol 1993;265:H755-H766.

**2.** Isenberg G, Klockner U. Calcium tolerant ventricular myocytes prepared by preincubation in a "KB medium". Pflugers Archiv 1982;395:6-18.

**3.** Yuill KH, Convery MK, Dooley PC, Doggrell SA, Hancox JC. Effects of BDF 9198 on action potentials and ionic currents from guinea-pig isolated ventricular myocytes. Br J Pharmacol 2000;130:1753-1766.

**4.** Zygmunt AC, Nesterenko VV, Rajamani S, Hu D, Barajas-Martinez H, Belardinelli L, Antzelevitch C. Mechanisms of atrial-selective block of Na^+^ channels by ranolazine: I. Experimental analysis of the use-dependent block. Am J Physiol 2011;301:H1606-H1614.
